# Post-quarantine anxiety and depression levels of COVID-19 positive patients, Northern Cyprus

**DOI:** 10.4314/ahs.v23i4.17

**Published:** 2023-12

**Authors:** Ufuk Kaya, Meryem Güvenir, Asli Aykac

**Affiliations:** 1 Cyprus International University, School of Health Sciences, Department of Nursing, Nicosia, Cyprus; 2 Cyprus Health and Social Sciences University, Faculty of Medicine, Department of Medical Microbiology, Morphou, Cyprus; 3 Near East University, Department of Biophysics, Nicosia, Cyprus

**Keywords:** anxiety, COVID-19, depression

## Abstract

**Background:**

The COVID-19 Pandemic, which started to be seen in Northern Cyprus (NC) as of March 2020, has affected the psychological mood of many people in our country as well as all over the world.

**Objectives:**

It was aimed to evaluate the post-illness anxiety and depression levels of people who were diagnosed with COVID-19 positive, completed the quarantine period and then received a negative PCR report.

**Methods:**

Through the questionnaire used in the study carried out between 1–30 April 2021, the sociodemographic characteristics of the participants and their personal experiences about COVID-19 were questioned and they were asked to answer the questions about the hospital anxiety and depression (HAD) scale.

**Results:**

The average score of the participants (n=120) in the HAD scale was determined as 11.66±5.90. According to the average scores of the scale, the anxiety and depression levels of the patients fall into the category of ‘11 and above abnormal’. The patients' general average scores of anxieties were 6.20±3.48 (normal) and depression was 5.46±3.55 (normal).

**Conclusion:**

Although it was determined that the HAD scores of the individuals from NC infected with COVID-19 were not high, most of them needed psychological support as they stated in their own statements.

## Introduction

The first COVID-19 infection case was uncovered in the Northern Cyprus (NC) on March 09, 2020. The total number of cases seen as of 25 April 2022 is 92,996. It was announced by the Ministry of Health that 235 of the cases resulted in death and the number of patients recovered was 91.464. The occurrence of different symptoms between individuals during the course of the disease may occur as well as the effects of the virus on the body after treatment may vary from person to person. In addition to the complications caused by the virus during the disease, there are many opinions about permanent complications after the illness 1, 2.

Individuals with anxiety disorders react highly disproportionately to the actual risk or danger present. Fear emerges as a result of perceived imminent threats in anxious person. As noted in the DSM-5 and ICD-10, fear or anxiety is pronounced, persistent, and disturbing 3, 4. In addition to existing physical or mental illnesses, the development of symptoms seen in anxiety disorders in individuals is among the most common conditions. For example, major depression and bipolar disorder are often accompanied by anxiety symptoms 5. Depression is a mood disorder characterized by psychomotor dysfunction, including an unwillingness to normal activities, and characterized by weakness and slow thinking 6. Anxiety can cause panic and fear in individuals, as well as depression in pandemic situations such as COVID-19 infection 7.

It is predicted that the fatal effects of the disease such as cough and shortness of breath may cause anxiety or depression in people due to the fear of losing their health and, accordingly, changes in their living standards such as nutrition and physical activity. Despite the smaller number of various psychological reports, results and statistics related to COVID-19 and its spread, it is an apparent fact that mental health problems may occur even after the epidemic and the health system will be seriously affected accordingly. In our study, we aimed to evaluate the presence of post-infection anxiety and depression in individuals who had COVID-19 infection, and to evaluate the relationship between possible changes in living standards and anxiety / depression in these individuals.

## Methods

### Participants

At the beginning of the questionnaire form presented in our study, in which participation was based on volunteerism, the participants were informed about the purpose and content of the research. The identity information of the survey participants was not recorded. Individuals over 18 years of age, without cognitive impairment and communication problems, without any disease that could cause depression, who was diagnosed with COVID-19 infection, were treated, recovered, and were negative for Polymerase Chain Reaction (PCR) were included in our study.

### Study design and HAD scale

The data were collected between 1-30 April 2021 using the information gathering form. The data collection form included questions questioning the characteristics of the individuals included in the study regarding the population structure and information on determining the change in living standards during and after the quarantine period. The “HAD scale”, which was not used for diagnostic purposes, was used to measure the mental states of the participants and to evaluate the symptoms of anxiety and depression and consisted of 14 items (both symptoms were evaluated with an equal number of questions, n=7)^8^-^13^. These items included, for example, for anxiety: ‘I suddenly feel panic’; for depression: ‘I lost interest in my appearance. The items on the scale were evaluated using a 4-point Likert scale with a scoring system ranging from 0-3 (from “didn't apply to me at all” to “applied to me very much or most of the time”). A score of “0-1” was considered not patient, “2” borderline patients and “3” seriously patient ill. In the evaluation of the total score, “0-7” points were considered normal, “8-10” borderline, and 11-21 points as abnormal 8. The total scores rage from 0 to 42. A higher score indicated a greater level of anxiety and depression.

### Study limitations

The research is limited to individuals aged 18 and over, living in the NC and agreeing to participate in the research. In addition, the number of participants was limited due to the intensity of the survey studies due to the epidemic. Another limitation of our study is that it was conducted within a certain time interval.

### Data analysis

Data were analysed using Statistical Package for Social Sciences (SPSS; ver. 22.0). Descriptive statistical methods such as percentage, standard deviation, frequency, mean and normal distribution were used in the evaluation of the research data. It was determined that the data showed normal distribution. The association between independent and dependent variables was determined using Pearson's chi-square test. The significance level was obtained with p – value (< 0.05).

## Results

In total, 64 (53.3%) participants were males and 56 (46.7%) were females. Majority of the participants belonged to 18–27 and 38-47 years age group (33.3 % and n=40, in both age groups) followed by 28–37 years (34.2%, n=24), 48-57 years and 58 ≤ years (6.7 %, n=8; in both age groups). Participants were residing in different regions of the country. Sixty-four (53.3 %) participants were residing in Nicosia, 16 (13.3 %) in Famagusta, 24 (20.0%) in Omorphou and 16 (13.3%) in Trimoko. Two of the three participants (66.7%) were married. Most of the participants had either two or no children (33.3%, n=40; in both married status). Sixty percent of the participants (n=72) were university graduates and 46.7% (n=56) were working as officers. All of the demographic data of the participants are presented in Table 1. Cronbach's *a* coefficients for this study were between 0.78 and 0.72 (for anxiety and depression, respectively).

**Table 1:** Descriptive characteristics of the participations (n=120)

Variable	n	%
**State of residence**		

Nicosia	64	53.3
Famagusta	16	13.3
Omorphou	24	20.1
Trimoko	16	13.3

**Age**		

18-27	40	33.3
28-37	24	20.0
38-47	40	33.3
48-57	8	6.7
58 ≤	8	6.7

**Gender**		

Female	56	46.7
Male	64	53.3

**Marital status**		

Married	80	66.7
Single	40	33.3

**Number of children**		

none	40	33.3
one	16	13.3
two	40	33.3
tree or more	24	20.1

**Educational status**		

Literate	24	20.0
Primary school	8	6.7
High school	16	13.3
University	72	60.0

**Employment Status**		

Unemployed	24	20.0
Employed	16	13.3
Officer	56	46.6
Self-employment	8	6.7
Housewife	8	6.7
Retired	8	6.7

**Total**	**120**	**100.0**

After the quarantine, 53.3% (n=64) of the patients had less contact with their family/relatives/colleagues/neighbours, 40% (n=48) received information about COVID-19 through TV and internet, 46.7% (n= 56) stated that they needed psychological support during the quarantine period, and 40% (n=48) stated that they increased their use of personal protective equipment more after the quarantine. 13.3% (n=16) of the patients reported fear of death during quarantine, 53.3% (n=64) feared being re-infected with the virus after quarantine, and 40% (n=48) reported disturbances in sleep patterns after quarantine (Table 2). 53.3% (n=64) of the participants declared that there was no change in their weight after quarantine. After the quarantine period, 20% (n=24) of the individuals increased their consumption of vegetables and vegetable dishes, while 13.3 (n=16) of the individuals reported that they changed their eating habits. When asked whether inactivity due to being closed in a room or a house during the quarantine period affects your health, one-third of the participants answered “yes”. 86.7% (n=104) of the patients took their medication regularly during the quarantine and none of the patients were re-infected with any variant of COVID-19 infection. 26.7% of the participants thought that some people they met might also have COVID-19.

**Table 2:** Participants' thoughts about living conditions during and after COVID-19 (n=120)

	n	%
**What is the situation that affects you the most about your life after quarantine?**		

I have never been affected by this situation.	32	26.7
My contact with family/relatives/colleague(s)/neighbour(s) has decreased	64	53.3
I moved away from entertainment and social life	8	6.7
I moved away from my family	16	13.3

**Where did you get information about COVID-19 during the quarantine?**		

TV and internet	48	40.0
Ministries and official organizations	32	26.7
Social media	32	26.7
Scientific research	8	6.6

**What support do you think you need in this process?**		

Psychological	56	46.7
Material	24	20.0
Health	16	13.3
None	24	20.0

**Which protection measures did you get more attention to after the quarantine?**		

Using personal protective equipment such as masks and gloves	48	40.0
Stay at home	32	26.7
Keeping social distance	24	20.0
Did not increase any protective measures	16	13.3

**Have you felt fear of death during quarantine?**		

Yes	16	13.3
No	104	86.7

**Have you had the fear of being infected with COVID-19 again after quarantine?**		

Yes	64	53.3
No	56	46.7

**Have you had any problems with your sleep pattern after quarantine?**		

Yes	48	40.0
No	72	60.0

**After the quarantine, have you started to consume medicinal plants to prevent the COVID-19 virus from**		

Yes	0	0
No	120	100.0

**Have your eating habits changed after quarantine?**		

Yes	16	13.3
No	104	86.7

**Has your weight changed after quarantine?**		

Decreased	32	26.7
None	64	53.3
Increased	24	20.0

**Which foods have you increased in your diet after the quarantine period?**		

Pastry food	16	13.3
Meat/meat products	8	6.7
Vegetables	24	20.0
Dessert	8	6.7
Snack	8	6.7
Fruit	24	20.0
None	32	26.6

**Has inactivity during quarantine affected your health?**		

Yes	40	33.3
No	80	66.7

**Have you used your medicines regularly during the quarantine?**		

Yes	104	86.7
No	16	13.3

**Have you been reinfected with COVID-19?**		

Yes	0	0
No	120	100.0

**Have you thought that some people you met might also have COVID-19?**		

Yes	32	26.7
No	88	73.3

**Total**	**120**	**100.0**

The average score of the participants in the HAD scale was determined as 11.66 ± 5.90. According to the average scores of the scale, the anxiety and depression levels of the patients fall into the category of ‘11 and above abnormal’. The patients' general average scores of anxieties were 6.20 ± 3.48 (normal) and depression was 5.46 ± 3.55 (normal) (Table 3).

**Table 3:** Distribution of the scores of the participants from the HAD scale (HAD) (n=120)

	Avg	SD	Minimum	Maximum
**Anxiety**	6.20	3.48	0	14
**Depression**	5.46	3.55	0	11
**General**	11.66	5.90	3	25

HAD: Hospital Anxiety and Depression; Avg: Average, SD: Standard deviation

Table 4 shows the comparison of the descriptive characteristics of the patients with the scale average scores. In the overall score averages of the scale; Living in Trimoko district (15.50±6.46), aged between 28-37 (14.33±7.47), female (13.14±6.45), single (11.80±4.63), a person with two children (14.40±6.36), high school education level (15.50±3.61) and employee (16.00±4.13) were determined as the variables with the highest average. Anxiety, depression and general average were found to be statistically significant according to residence and employment status (*p* = 0.000). Anxiety and general average were found to be statistically significant according to age (*p* = 0.000 and *p* = 0.009), gender (*p* = 0.007 and p =0.004) and number of children (*p* = 0.000 and *p* = 0.001). Anxiety and depression were found to be statistically significant according to marital status (*p* = 0.007 and *p* =0.001) and educational level (*p* = 0.003 and *p* = 0.000).

**Table 4:** Comparison of the descriptive characteristics of the participants with the scale average scores (n=120)

Introductory Feature	Anxiety	Depression	General	

	Avg ± SD	Avg ± SD	Avg ± SD	
**Region**				

Nicosia	5.12±3.20	7.12±3.39	12.25±6.30	
Famagusta	4.50±1.54	0.50±0.51	5.00±1.03	
Omorphou	7.66±2.09	4.33±5.68	12.00±3.82	
Trimoko	10.00±4.13	5.50±0.51	15.50±4.64	

	***0.000*** *	***0.000*** *	***0.000*** *	***p***

**Age**				

18-27	5.20±2.67	5.40±3.65	10.60±5.18	
28-37	8.33±4.59	6.00±4.17	14.33±7.47	
38-47	6.80±3.29	6.00±3.44	12.80±5.70	
48-57	3.00±0.00	5.00±0.00	8.00±0.00	
≥ 58	5.00±0.00	2.00±0.00	7.00±0.00	

	***0.000*** *	**0.070**	***0.009*** *	***p***

**Gender**				

Female	7.28±3.64	5.85±3.94	13.14±6.45	
Male	5.25±3.05	5.12±3.16	10.37±5.08	

	***0.007*** *	**0.310**	***0.004*** *	***p***

**Marital status**				

Married	6.90±3.64	4.70±3.76	11.60±6.47	
Single	4.80±2.67	7.00±2.48	11.80±4.63	

	***0.007*** *	***0.001*** *	0.858	p

**Number of Child**				

None	5.20±2.67	5.40±3.65	10.60±5.18	
One	3.50±0.51	4.50±3.61	8.00±4.13	
Two	8.80±3.96	5.60±3.30	14.40±6.36	
Three or more	5.33±2.09	6.00±3.82	11.33±5.55	

	***0.000*** *	0.660	***0.001*** *	***p***

**Education Status**				

Reader-Writer	4.66±3.37	7.33±2.92	12.00±6.01	
Primary School	3.00±0.00	5.00±0.00	8.00±0.00	
High School	6.00±2.06	9.50±1.54	15.50±3.61	
University	7.11±3.59	4.00±3.28	11.11±6.23	

	***0.003*** *	***0.000*** *	0.054	***p***

**Working Status**				

Not work	4.66±3.37	7.33±2.92	12.00±6.01	
Employee	7.00±3.09	9.00±1.03	16.00±4.13	
Officer	7.85±3.25	4.85±3.63	12.71±5.75	
Independent worker	2.00±0.00	1.00±0.00	3.00±0.00	
Housewife	3.00±0.00	5.00±0.00	8.00±0.00	
Retired	5.00±0.00	2.00±0.00	7.00±0.00	

	***0.000*** *	***0.000*** *	***0.000*** *	***p***

* *p<0.05*

Avg: Average, SD: Standard deviation

Table 5 shows the comparison of the medical condition-related characteristics of the patients with the scale average scores. In the overall score averages of the scale; Having reduced contact with family/relatives/colleagues/neighbours (12.62±5.98), learning information about COVID-19 through scientific research (20.00±0.00), not needing any support in the process (13.33±2.09), post-quarantine personal protective equipment who pay attention to the precautions (14.00±5.56), feel fear of death during the quarantine process (13.50±5.68), do not experience the fear of re-infecting the virus (12.28±5.02), have sleep problems after quarantine (12.66±7.28), do not change their eating habits after quarantine (11.69± 5.39), losing weight (14.66±7.70), consuming more sweets and products (19.00±0.00), thinking that inactivity affects their health (16.80±2.82), using their medications regularly (12.15±5.81) and not thinking that people they see may have COVID-19 (12.09±5.74) patients' scale score averages were found to be higher.

It was determined that the scale average scores obtained from the questions asked about the medical conditions of the patients significantly affected the anxiety, depression and general average score scores in the following questions: What is the situation that affects you the most about your life after quarantine; Where did you get information about COVID-19 during the quarantine; Which measures did you pay more attention to after the quarantine; How has your weight changed after quarantine; Which foods did you increase in your diet after the quarantine process; Has inactivity during the quarantine affected your health (Table 5; *p* < 0.000 - 0.005).

It was determined that the anxiety scale average score obtained from the questions asked about the medical conditions of the patients was significantly affected only in the following question: Have you thought that some of the people you see might have COVID-19? (*p* < 0.05). It was determined that the depression scale average score obtained from the questions asked about the medical conditions of the patients was significantly affected only in the following question: Have you felt fear of death during the quarantine? (Table 5, *p* < 0.05).

**Table 5:** Comparison of patients' medical condition-related characteristics with the scale average scores (n=120)

	Anxiety	Depression	General	

	Avg ± SD	Avg ± SD	Avg ± SD	
**What is the situation that affects you the most about your life after quarantine?**				

I' m not impressed at all	6.75±2.42	5.25±2.81	12.00±3.29	
My contact with family/relatives/ colleagues/neighbours has decreased	6.62±3.96	6.00±3.26	12.62±5.98	
I moved away from entertainment and social life	2.00±0.00	1.00±0.00	3.00±0.00	
I moved away from my family	5.50±2.58	6.00±5.16	11.50±7.74	

	***0.000*** *	***0.003*** *	***0.000*** *	***p***

**Where did you get information about COVID-19 during the quarantine?**				

TV and internet	6.33±3.58	6.83±3.05	13.16±5.39	
Ministries and official organizations	6.25±1.95	6.75±3.68	13.00±5.47	
Social Media	4.00±1.60	2.00±1.90	6.00±1.90	
Scientific Research	14.00±0.00	6.00±0.00	20.00±0.00	

	***0.000*** *	***0.000*** *	***0.000*** *	***p***

**What support do you think you need in this process?**				

Psychological Support	6.42±4.27	4.85±4.05	11.28±7.12	
Financial Support	4.66±2.54	6.66±4.28	11.33±6.68	
Health Support	5.00±2.06	6.00±1.03	11.00±3.09	
I didn't need any support	8.00±1.66	5.33±2.09	13.33±2.09	

	0.235	0.082	0.650	***p***

**Which measures did you pay more attention to after the quarantine?**				

Using personal protective equipment such as masks and gloves	7.50±3.63	6.50±3.12	14.00±5.56	
Stay at home	4.25±3.39	5.75±3.75	10.00±6.85	
Keeping Social Distance	433±127	3.00±3.63	7.33±3.47	
I did not increase the measures	9.00±1.03	5.50±2.58	14.50±1.54	

	***0.000*** *	***0.001*** *	***0.000*** *	***p***

**Have you felt fear of death during the quarantine?**				

Yes	5.50±2.58	8.00±3.09	13.50±5.68	
No	6.30±3.59	5.07±3.46	11.38±5.91	

	0.619	***0.003*** *	0.137	***p***

**Have you had the fear of being infected again after the quarantine?**				

Yes	5.62±2.61	5.50±4.39	11.12±6.57	
No	6.85±4.19	5.42±2.27	12.28±5.02	

	0.062	0.735	0.176	***p***

**Have you had sleep patterns and problems after quarantine?**				

Yes	7.00±4.12	5.66±3.94	12.66±7.23	
No	5.66±2.88	5.33±3.28	11.00±4.77	

	0.300	0.730	0.228	***p***

**Have your eating habits changed after quarantine?**				

Yes	6.00±4.13	5.50±4.64	11.50±8.77	
No	6.23±3.39	5.46±3.38	11.69±5.39	

	0.804	0.804	0.804	***p***

**How has your weight changed after quarantine?**				

Increase	6.00±2.48	6.25±3.75	12.25±6.15	
Not Changed	5.25±2.83	5.00±3.34	10.25±4.47	
Decrease	9.00±4.64	5.66±3.76	14.66±7.70	

	***0.001*** *	0.243	***0.004*** *	***p***

**Which foods did you increase in your diet after the quarantine process?**				

Pastry	7.50±0.51	7.50±0.51	15.00±1.03	
Meat and meat products	2.00±0.00	1.00±0.00	3.00±0.00	

Vegetables and vegetable dishes	2.66±2.09	3.66±1.27	6.33±1.73	
Dessert	8.00±0.00	11.00±0.00	19.00±0.00	
Types of junk food	6.00±0.00	5.00±0.00	11.00±0.00	
Fruit and berry foods	10.33±2.92	6.66±3.37	17.00±3.00	
None	5.75±2.72	4.75±4.39	10.50±6.32	

	***0.000*** *	***0.000*** *	***0.000*** *	***p***

**Has inactivity during the quarantine affected your health?**				

Yes	8.40±1.37	8.40±3.11	16.80±2.82	
No	5.10±3.69	4.00±2.77	9.10±5.35	

	***0.000*** *	***0.000*** *	***0.000*** *	***p***

**Have you used your medicines regularly during the quarantine?**				

Yes	6.46±3.53	5.69±3.57	12.15±5.81	
No	4.50±2.58	4.00±3.09	8.50±5.68	

	***0.025*** *	0.082	***0.013*** *	***p***

**Have you thought that some of the people you see might have COVID-19?**				

Yes	5.25±3.16	5.25±3.24	10.50±6.28	
No	6.54±3.54	5.54±3.67	12.09±5.74	

	***0.035*** *	0.567	0.182	***p***

* *p<0.05*

Avg: Average, SD: Standard deviation

## Discussion

In our study, it was aimed to determine the presence of anxiety and depression in people who had COVID-19 infection (received COVID-19 treatment, completed the quarantine process and became negative), as well as to evaluate the changes in living standards due to a positive diagnosis of COVID-19 in these individuals. When we evaluate the volunteers participating in our study, we see that the average age of our sample group is low, the male gender is high, the rate of comorbidities is low, and the infection consists of individuals with a mild course.

Epidemics cause mental health problems as well as physical health problems in society. Therefore, it is necessary to see the pandemic not only as a health crisis, but also as an infectious disease that threatens the mental health of society. At the beginning of the pandemic, our knowledge, including the transmission routes of the virus, its symptoms, the diseases it causes and its treatment, was very limited. Over time, our knowledge about the physical effects and process of the disease began to increase. However, the number of studies on the impact of the pandemic on public MD has not reached sufficient levels yet. However, even if the pandemic is over, when we return to our normal lives, the psychological effects caused by the epidemic will probably continue for a long time 14.

In the article published in 2020, the findings of the studies on the mental effects of quarantine in epidemic diseases were reviewed, and the most common mental complaints and diseases, risk factors and risk groups during the current pandemic period were re-evaluated. It has been reported that feelings of fear, irritability, sadness and guilt are common during the quarantine period in people who have been quarantined for coming into contact with a person infected with the SARS virus. Among the long-term effects of the quarantine, it was emphasized that behavioral changes such as avoidance of crowds and extreme careful hand washing were observed, so some people could not return to normal life for months 15.

Studies on how long the negative effects of mental health problems developed due to quarantine last are limited, and the studies are mostly on health workers. In studies on health workers; Three years after the SARS epidemic, an increase in the prevalence of alcohol abuse and addiction among healthcare workers was reported. In another study, even three years after quarantine, hospital workers were found to be under quarantine in the past as a risk factor for the development of PTSD 15. According to the data of our study; reported that 53.3% of the people who participated in the study reduced their contact with their family/ relatives/colleagues/neighbours. In addition, the participants whose contact with their family/ relatives/colleagues/neighbours decreased, were found to have higher average scores than the other group.

Nutrition is one of the most important issues that need to be emphasized today. The use of nutrients in the body for the purpose of sustaining life and protecting health is defined as nutrition. In other words, it is a behaviour that aims to take the nutrients needed by the body in sufficient quantities and at the appropriate time in order to protect health and increase the quality of life 16. Appropriate nutritional therapy is recommended for many diseases, including acute, chronic and infectious diseases. For this reason, it is very important to comply with the principles of healthy eating during quarantine and normal times 17. According to the data of our study; reported that 36.7% of the participants did not change their eating habits after the quarantine and 53.3% did not experience weight change, but 20% of them increased their vegetable and vegetable meals and fruit and fruit drinks in their post-quarantine diet. However, participants whose eating habits did not change after quarantine, whose weight decreased, who consumed more sweets and products were found to have higher average scores.

After the completion of the treatment received at the hospital by the participants with symptoms of COVID-19, they were quarantined in a dormitory or hotel used as a quarantine center until the PCR results became negative. Participants without symptoms were quarantined directly in campuses designated as quarantine centers. It was determined that the anxiety and depression scores of the individuals participating in the study were 6.2±3.48 and 5.4±3.53. Turan et. al. reported anxiety level was 45.1% and depression level was 23.6% for Turkish Society during pandemic 18. Additionally, Turan et. al. reported that, 44.6% of participants scored above anxiety and 68.2% scored above depression cut-off points according HAD scale for Turkish health-workers 18.

In a study in which patients with the SARS epidemic were evaluated psychiatrically during the early recovery period, depressive complaints were found in 18% of the participants and anxiety symptoms in 14% 19. Likewise, another study conducted during the early recovery period of the SARS epidemic found that 35% of those who had the disease had symptoms of depression or anxiety disorder 20. When compared with the results of these two studies, the frequency of anxiety or depressive symptoms in our study group was found to be quite low compared to the results of other studies. In a study conducted with 7,143 students after the COVID-19 outbreak in China, 0.9% of the participants stated that they experienced intense, 2.7-21.3% moderate-mild anxiety symptoms, when looking at the psychological effects of the epidemic on individuals in 1,210 participants; They declared that they spend most of the day at home instead of going out due to the pandemic and they are afraid of their family members getting COVID-19 21, 22. They stated that 16.5% of the participants showed moderate to severe depression and 28.8% showed moderate to severe anxiety symptoms reported that 32.4% of the participants showed symptoms of anxiety and 44.1% of depression in a study conducted on the internet with 3,550 individuals in Spain, where COVID-19 is widely seen 22, 23. Rossi et al. (2020) conducted a survey with 18.147 participants online to examine the effect of the pandemic and quarantine process on the psychological health of individuals in Italy, and as a result of the study, 37% of the participants had PTSD, 20.8% anxiety, 17.3% depression 24. They reported that 7.3% had sleep problems and 22.9% had adjustment disorder symptoms. According to the data of our study; Participants who felt fear of death during quarantine and had sleep problems after quarantine had higher average scores on the scale 24.

Although there are studies in the literature evaluating the clinical symptoms caused by the COVID-19 epidemic and the psychological effects with healthcare professionals, the number of studies on social psychological effects and the changes in living standards caused by these effects is not yet sufficient. However, the results of the few studies summarized above emphasize that there is a positive correlation between epidemic diseases and mood disorders. As in natural disasters, an increase in the frequency of mental disorders (MD) such as major depression, PTSD, generalized anxiety disorder, obsessive-compulsive disorder is predicted in the community during and after the epidemic. In addition, it has been revealed that the risk of developing a MD in individuals increases the risk of developing a MD if the quarantine period lasts longer than 10 days ^25^. Although COVID-19 was clinically mild in the participants in our study, the loneliness they experienced during the isolation period, being away from their loved ones, uncertainty, the feeling of being restricted in their freedom, the obligation to follow some rules in terms of public health may have led to the development of symptoms of anxiety and depression in the individuals included in our study. Although COVID-19 was clinically mild in the participants in our study, the loneliness they experienced during the isolation period, being away from their loved ones, uncertainty, the feeling of being restricted, the obligation to follow some rules in terms of public health may have led to the development of symptoms of anxiety and depression in the future. According to the data of our study, it was concluded that 46.7% of the participants needed psychological support. However, they reported that 86.7% of the participants did not feel fear of death during the quarantine period.

Our study has several important limitations. In future research, interviewing on the internet and filling in the scales may provide a larger sample. Another limitation of our study was that the volunteers generally consisted of young individuals with mild disease and no diagnosis of additional disease. Different results could have been obtained in a study with a larger sample, in which the disease progressed more severely, in the advanced age group and in people with a diagnosis of additional disease.

## Data Availability

Ethical restrictions have been imposed on the data in this study to protect the confidentiality of participant information. Interested researchers may submit queries related to data access to the corresponding author.
